# The Role of Emotional Landmarks on Topographical Memory

**DOI:** 10.3389/fpsyg.2017.00763

**Published:** 2017-05-10

**Authors:** Massimiliano Palmiero, Laura Piccardi

**Affiliations:** ^1^Neuropsychology Unit, I.R.C.C.S. Fondazione Santa LuciaRome, Italy; ^2^Department of Applied Clinical and Biotechnological Sciences, University of L’AquilaL’Aquila, Italy; ^3^Department of Life, Health and Environmental Sciences, University of L’AquilaL’Aquila, Italy

**Keywords:** navigation, wayfinding, landmark-based navigation, memory, arousal, valence, egocentric reference frame, allocentric reference frame

## Abstract

The investigation of the role of emotional landmarks on human navigation has been almost totally neglected in psychological research. Therefore, the extent to which positive and negative emotional landmarks affect topographical memory as compared to neutral emotional landmark was explored. Positive, negative and neutral affect-laden images were selected as landmarks from the International Affective Picture System (IAPS) Inventory. The Walking Corsi test (WalCT) was used in order to test the landmark-based topographical memory. Participants were instructed to learn and retain an eight-square path encompassing positive, negative or neutral emotional landmarks. Both egocentric and allocentric frames of references were considered. Egocentric representation encompasses the object’s relation to the self and it is generated from sensory data. Allocentric representation expresses a location with respect to an external frame regardless of the self and it is the basis for long-term storage of complex layouts. In particular, three measures of egocentric and allocentric topographical memory were taken into account: (1) the ability to learn the path; (2) the ability to recall by walking the path five minutes later; (3) the ability to reproduce the path on the outline of the WalCT. Results showed that both positive and negative emotional landmarks equally enhanced the learning of the path as compared to neutral emotional landmarks. In addition, positive emotional landmarks improved the reproduction of the path on the map as compared to negative and neutral emotional landmarks. These results generally show that emotional landmarks enhance egocentric-based topographical memory, whereas positive emotional landmarks seem to be more effective for allocentric-based topographical memory.

## Introduction

Navigation is essential for humans in order to adapt to the living environment and get successful mastery of daily life. Memory certainly plays a crucial role on navigation. People spend a lot of time recalling and figuring a route or a shortcut out to reach a place. Of course, well-known paths are remembered easily and require less working memory capacities (Montello, 2009; Nori and Piccardi, 2011). On the contrary, unknown paths require more attention and greater working memory capacities (Montello, 2009). Interestingly, when people recall a pathway from memory a specific spatial memory system is used (e.g., Piccardi et al., 2010). This system is called topographic memory and involves not only visuospatial information, but also vestibular and proprioceptive information relative to the whole-body movements, as well as a continuous change of the person’s point of view that implies an active updating of the mental representation and the person’s position in the environment (e.g., Piccardi et al., 2008, 2013). This system was found to be separated by the standard visuo-spatial memory at neural level in normal subjects (Nemmi et al., 2013), as well as in different neuropsychological disorders, such as brain-damaged patients (Piccardi et al., 2011a) or in patients with Alzheimer’s disease at the early stages (Bianchini et al., 2014).

Topographical memory is widely supported by object location memory, which has a role in maintaining a coherent and meaningful representation of the visual world, as well as in providing a platform from which directional information can be generated (Postma and De Haan, 1996; Gronau et al., 2008; Postma et al., 2008). For example, remembering the position of a landmark implies the capacity to process landmark-identity information (what), landmark-position (where), and the binding of what and where information (Moscovitch et al., 1995). In general, ‘a landmark is a salient environmental cue working as a spatial reference’ (Nico et al., 2008, p. 1898). They refer to any feature of the environment that is recognizable and serve as spatial reference, such as edges or barriers, rivers, squares, lakes, particular buildings, city monuments and so forth. For example, many people use the Coliseum as reference point to get familiarized with Rome and better navigate the city. Therefore, landmarks play a key role on the construction of mental representations of the navigational environment (Raubal and Winter, 2002), given that perceptual representations of the spatial scene in topographical memory encompass landmarks with their features.

In order to be effective for navigation landmarks must be structurally (with a prominent spatial location – Raubal and Winter, 2002), visually (with a particular size, color, and shape) and semantically (depending on cultural, personal and historical influences) relevant (Caduff and Timpf, 2008). In addition, landmarks can also be emotionally loaded (Balaban et al., 2017), enhancing (positively or negatively) the ability to construct a cognitive map. In other words, the emotional salience of the landmark rather than the individual’s emotional state can be relevant as an aid for navigation. According to Gartner (2012) emotions that are linked to specific landmarks may facilitate navigation when the cognitive state is overloaded, as well as enhance the process of cognitive mapping at any moment. In this direction, Kitchin (1996) developed a conceptual model of the environment–behavior interaction. This model includes three basic components: ‘real world’, representing the environment people interact with; ‘working memory’, that encompasses different filters, including the current emotional state filter which works in partnership with the perceptual context; ‘long term memory’, that contains both records of situations within a time framework and an information store. Therefore, in this model emotions also act as filters in the process of building up a cognitive map.

Although emotions were found to influence topographical memory in real environments (e.g., Palmiero et al., 2015, 2016), the literature that specifically addressed the issue of the effectiveness of emotional landmarks in navigation and wayfinding is extremely scarce (Gartner, 2012). To the best of our knowledge, using virtual environments only Balaban et al. (2017) specifically investigated the role of affect-laden landmarks on wayfinding and recognition with respect to neutral landmarks. Results showed that negatively laden landmarks led to better wayfinding and recognition performance than neutral and positively laden landmarks. Furthermore, they found that negative associations are better remembered over time than positive and neutral associations. According to Balaban et al. (2017), the affective valence of the landmarks enhances wayfinding performance.

Thus, in the present study, the aim was to explore the extent to which topographical memory can be influenced by emotional landmarks. Two different frame of references were considered: “allocentric” (world-centered) and “egocentric” (body-centered) frames of references (Burgess, 2006, 2008; Arleo and Rondi-Reig, 2007). Indeed, individuals may locate environmental objects by a) referring to their own position, namely Egocentric frame of reference or by b) referring to the spatial and configurational properties of such objects, namely Allocentric frame of reference (Galati et al., 2000). To move in the environment or to provide spatial direction, individuals combine spatial frames of reference with spatial relations (Ruotolo et al., 2011). For example, “the street closer to me/on my left” (egocentric) or “the flower shop closer to the church/on the right of the church” (allocentric). The existence of these two frames of reference is confirmed by fMRI studies (Hartley et al., 2003; Iaria et al., 2007; Latini-Corazzini et al., 2010; Sulpizio et al., 2013, 2016). Specifically, egocentric navigation involves areas including the parahippocampal place area (PPA), precuneus and cuneus, inferior parietal lobe and retrosplenial cortex (RSC) (see also Byrne et al., 2007). Instead, allocentric navigation is mainly supported by areas containing place cells (hippocampus) and grid cells (entorhinal cortex) (Maguire et al., 1998; Byrne et al., 2007).

In addition, egocentric and allocentric frames of reference yield to different types of spatial knowledge, such as Route and Survey knowledge, respectively. Route knowledge is characterized as knowledge of spatial layout from the perspective of a ground-level observer. Survey knowledge is characterized by an external perspective, such as a bird’s-eye view, which allows for direct access to the global spatial layout (Shelton and Gabrieli, 2002). Evidence for a distinction between these types of spatial knowledge comes from behavioral (Siegel and White, 1975; O’Keefe and Nadel, 1978; Montello, 1998; Tversky, 2000) and fMRI studies (e.g., Mellet et al., 2000; Shelton and Gabrieli, 2002; Boccia et al., 2016). In particular, Shelton and Gabrieli (2002) found that survey encoding recruits areas also recruited by route encoding, but with greater activation in the inferior temporal cortex and in the posterior superior parietal cortex. Furthermore, only route encoding recruited the medial temporal lobe structures, anterior superior parietal cortex and postcentral gyrus.

Therefore, with this in mind, topographical memory, as measured by the Walking Corsi Test (WalCT), was explored using positive, negative or neutral emotional landmarks in terms of both egocentric and allocentric references of frames. Three aspects of topographical memory were assessed: learning a path of eight steps (egocentric frame); delayed recall (five minutes later) of the previously acquired path (egocentric frame); reproduction of the path on the outline of the WalCT (allocentric frame). This is the first study that explored such an emotional landmark-based topographical memory taking into account for the two different systems of spatial frame of references (egocentric vs. allocentric coordinates).

## Materials and Methods

### Participants

For this study 75 College students were recruited from the “Department of Life, Health and Environmental Sciences”, University of L’Aquila, Italy. Participants were divided in three groups according to the type of landmarks they were exposed while performing on the topographical memory tasks:

- 25 participants for the positive landmark group (PLG): 11 females and 14 males (mean age = 23.64 ± 3.34; age range = 19–30 years);
- 25 participants for the negative landmark group (NLG): 14 females and 11 males (mean age = 22.36 ± 1.5; age range = 21–25 years);
- 25 participants for the neutral landmark group (NeuLG): 15 females and 10 males (mean age = 22.36 ± 1.98; age range = 19–26 years).

All participants were healthy and without neurological and/or psychiatric disorders; no problem with alcohol or drug addiction was reported. All participants had normal or corrected to normal (soft contact lenses or glasses) vision. Moreover, all participants performed the Familiarity and Spatial Cognitive Style scale (FSCS; Piccardi et al., 2011b) which includes 22 self-referential statements about various aspects of environmental spatial cognition. The FSCS was used to exclude participants with self-declared topographical orientation disorders. None of the participants showed the presence of navigational deficits or developmental topographical disorientation (see Iaria et al., 2005, 2009; Bianchini et al., 2010). All participants filled out the anamnesis questionnaire aimed at collecting demographic, health information (e.g., trauma, surgeries, psychiatric and neurological disorders) and alcohol/drugs assumption information. The written informed consent was signed by everyone. The study was designed in accordance with the latest version of the Declaration of Helsinki and was approved by the local ethical committee.

### Materials and Procedure

#### Images from The International Affective Picture System (IAPS) Inventory

In order to get positive, negative and neutral emotional landmarks, 9 affect-laden images (30 cm × 30 cm) were taken from the Images from The International Affective Picture System (IAPS) Inventory (Bradley and Lang, 2007; Lang et al., 2008), that includes standardized colored photographs representing three categories of emotional stimuli (positive, negative and neutral), being scored in terms of valence (ranging from pleasant to unpleasant), arousal (ranging from calm to excited) and dominance (ranging from in control to dominated). In the present study, images were differentiated according to their valence and arousal, which are the two fundamental aspects of emotionality (Russel, 1980), according to the original scores (Lang et al., 2008), as follows:

- 3 positive emotional images with high valence (mean = 8.02, standard deviation (*SD*) = 0.25) and high arousal (mean = 6.21, *SD* = 0.47), namely the images of ‘beach’, ‘skier’ and ‘sailing’;
- 3 negative emotional images with low valence (mean = 1.55, standard deviation = 0.12) and high arousal (mean = 6.82, *SD* = 0.6), namely the images of ‘face mutilated’, ‘soldier’ and ‘dog’;
- 3 neutral emotional images both in terms of valence (mean = 6.03, *SD* = 1.18) and arousal (mean = 3.37, *SD* = 0.22), namely the images of ‘parrots’, ‘cow’, and ‘man’.

Positive and negative emotional images were purposefully selected with comparable arousal but different valence in order to differentiate these two categories of stimuli only in terms of valence. In addition, neutral emotional images were selected following valence and arousal values indicated in Nowicka et al.’s (2011) study.

#### Positive and Negative Affect Schedule (PANAS)

In order to evaluate how the participant was feeling ‘right now’, the Italian version (Terracciano et al., 2003) of the Positive and Negative Affect Schedule (PANAS) (Watson et al., 1988) was used. This schedule consisted of 10 positive adjectives and 10 negative adjectives that were scored using a 5-point Likert scale, ranging from 1 (very slightly or not at all) to 5 (extremely).

#### The Walking Corsi Test (WalCT)

In order to measure the topographical memory, the WalCT (Piccardi et al., 2008, 2013) was used. This test consisted of a larger version of the Corsi block tapping test (CBT - Corsi, 1972) (3 m × 25 m; scale 1:10 of the CBT). Nine squares (30 cm × 30 cm) were placed on the floor of an empty room in the same position as in the standard CBT. In addition, one more square was placed half meter from the line determining the perimeter of the walking area. The location of the starting position was decided in a pilot study that different points of view did not influence performance on the WalCT (as reported in Piccardi et al., 2008).

**FIGURE 1:**
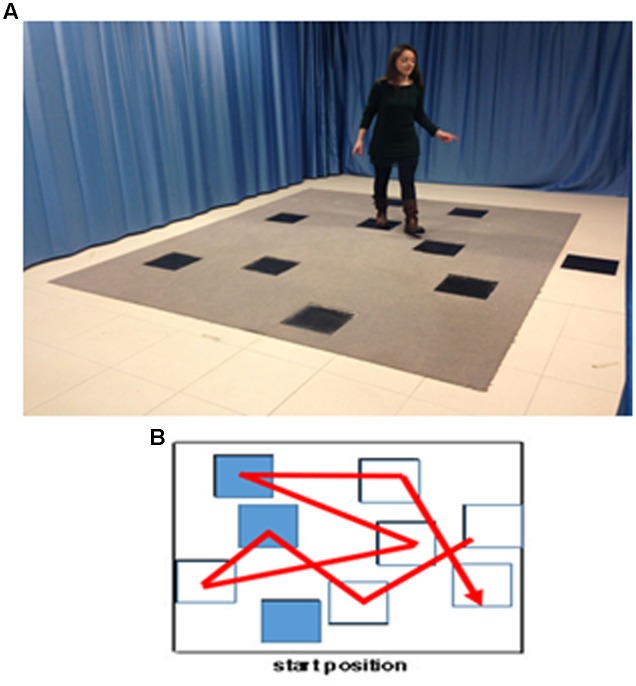
**The landmark-based navigational memory task**. The eight-square path was designed in order to let participants move through the squares, as showed by the red line. **(A)** Experimental set-up. Written informed consent was obtained from the subject represented in the figure for publication of this experiment. A copy of the written consent is available for review by the Editor-in-Chief of this journal. **(B)** Disposition of the positive, negative and neutral landmarks through the path.

Then, the WalCT with landmarks was set up in three different conditions (positive, negative and neutral) using the 9 affect-laden images previously selected from the IAPS Inventory. These images were placed on the squares of the WalCT, in the same position for all landmark conditions in order to obtain comparable intersections among squares, involving the same spatial distances with respect to the path to learn. (See **Figure 1** for the complete set up of the landmark-based navigational memory task).

The WalCT consisted in different tasks, as follows:

##### (i)First egocentric topographic memory task – learning of the sequence

The experimenter showed an eight-square path by walking on squares at a rate of one square per 2 s. Participants were instructed to learn the path. The eight-square path was the same for all conditions. The learning criterion was reached if participants reproduced the correct path three times in a row. Thus, if participants failed in reproducing the path, the experimenter showed it again (max number of trials = 18) until the learning criterion or the max number of trials were reached. The learning score is calculated as follows: one point is attributed for each square correctly touched in the sequential order showed by the experimenter, until the criterion was reached; then, eight points are summed for each of the remaining trials (up to the 18th; maximum total score: 144). For example, if the participants reached the learning criterion by the third repetition, that is with no failing, they obtained a score of 8 squares X 3 = 24, plus 8 squares X 15 = 120 for the remaining trials. Thus, they obtained a total score of 144, which was the maximum score.

##### (ii)Egocentric topographic memory task – delayed recall

Five minutes later, the experimenter asked participants to reproduce by walking the previously learned eight-square path. The delayed recall score was calculated on the basis of the number of squares correctly reproduced (maximum score = 8).

##### Allocentric topographic memory task – drawing the learned sequence on the map

**FIGURE 2:**
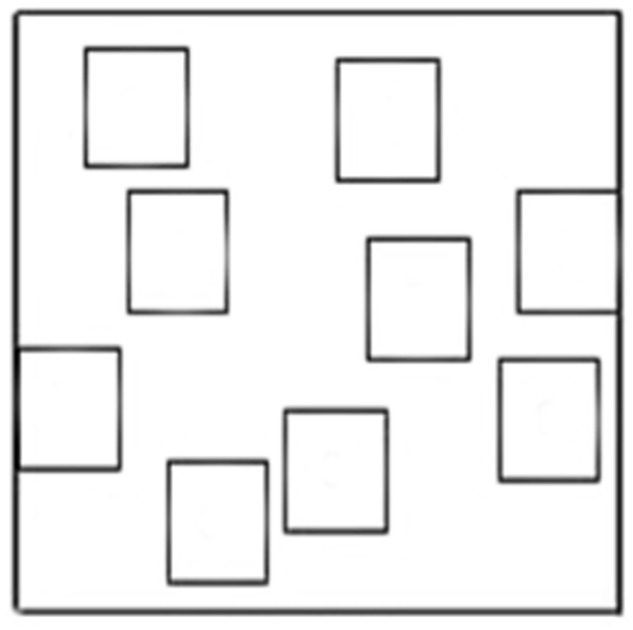
**Outline of the Walking Corsi Test**.

Participants were asked to use a felt tip marker to retrace the eight-square path on the outline representing the configuration of the WalCT (see **Figure 2**). Also for this task the total score was the number of squares correctly reproduced (maximum score = 8). At this point in time, participants were asked to fill out the PANAS for the second time.

##### Recognition landmark task

Participants were asked to recognize the three images used as positive, negative or neutral emotional landmarks mixed among three distractors comparable in terms of valence and arousal (for positive stimuli, valence: *mean* = 8.05 ± 0.25, arousal: *mean* = 6.11 ± 0.52; for negative stimuli, valence: *mean* = 1.71 ± 0.17, arousal: *mean* = 7.11 ± 0.21; for neutral stimuli, valence: *mean* = 5.95 ± 0.55, arousal: *mean* = 3.43 ± 0.33). Participants recognized all landmarks used regardless the category of stimuli.

In short, firstly, participants were given the basic instructions to run the experiment. They were randomly assigned to one of the following groups according to the valence of landmarks used: positive landmark group (PLG), negative landmark group (NLG), or neutral landmark group (NeuLG). Afterwards, to check the individual’s mood manipulation, participants filled out the PANAS for the first time. Next, participants performed on the three topographical memory tasks (learning, delayed recall and reproduction or the eight-square sequence) in positive, negative or neutral emotional landmark condition. Then, participants filled out the PANAS for the second time. Finally, they performed on the recognition landmark task.

## Results

### Individual’s Mood Manipulation Check

The PANAS was used to control for individual’s mood changes. Following the procedure used by Phillips et al. (2002), mood scores at both the first and the second administration of the PANAS were obtained by subtracting the total negative affect score (computed summing the scores for each of the 10 negative adjectives) from the positive affect score (computed summing the scores for each of the 10 positive adjectives). Then, comparing mood scores at the first administration (baseline) to mood scores at the second administration (after the completion of the WalCT) in terms of group conditions (positive, negative and neutral landmarks) no significant results were found: no main effects of ‘group’ [*F*(2,72) = 1.5743, *p* = 0.21, partial η^2^ = 0.042]; and ‘time’ [*F*(1,72) = 1.4298, *p* = 0.24, partial η^2^ = 0.019]; no interaction effect of ‘group and time’ [*F*(2,72) = 0.44, *p* = 0.65, partial η^2^ = 0.012]. No significant results were obtained even considering separately positive (no main effects of ‘group’ [*F*(2,72) = 2.513, *p* = 0.09, partial η^2^ = 0.065], ‘time’ [*F*(1,72) = 0.029, *p* = 0.87, partial η^2^ = 0.00] and interaction effect of ‘group and time’ [*F*(2,72) = 0.812, *p* = 0.45, partial η^2^ = 0.022]) and negative affect (no main effects of ‘group’ [*F*(2,72) = 0.0928, *p* = 0.91, partial η^2^ = 0.003], ‘time’ [*F*(1,72) = 3.0565, *p* = 0.08, partial η^2^ = 0.041] and interaction effect of ‘group and time’ [*F*(2,72) = 0.038, *p* = 0.96, partial η^2^ = 0.001]). These results indicated that any effect on topographical memory performance would be due to the emotional landmarks rather than to the participants’ mood changes.

### Learning of the Eight-Square Sequence

Descriptive statistics for this measure follows: *Mean* = 130.8; *SD* = 9.11; *SE* = 1.05; Min = 103–Max = 144.

**FIGURE 3:**
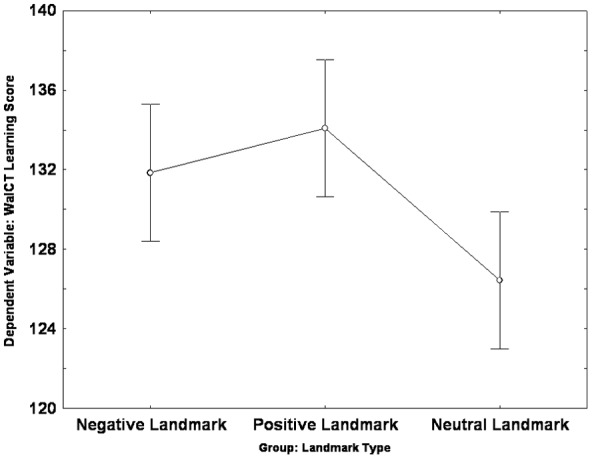
**Landmark group differences in the learning score of the Walking Corsi Test**. The error bars represent the standard errors of the means (confidence interval = 0.95).

The Univariate ANOVA carried out on the learning score revealed an effect of group [*F*(2,72) = 5.17, *p* = 0.008, partial η^2^ = 0.126]: *Post hoc* analysis (LSD: *p* < 0.05) showed that both the PLG (*mean* = 134.08; *SE* = 1.73) and the NLG (*mean* = 131.84; *SE* = 1.73) scored higher than the NeuLG (*mean* = 122.44; *SE* = 1.73); no difference was found between the PLG and the NLG (See **Figure 3**).

### Delayed Recall

Descriptive statistics for this measure follows: *Mean* = 7.76; *SD* = 1.11; *SE* = 0.13; Min = 0–Max = 8.

The Univariate ANOVA carried out on the delayed recall score showed no difference [*F*(2,72) = 0.126, *p* = 0.88, partial η^2^ = 0.003] among the PLG (*mean* = 7.84; *SE* = 0.23), the NLG (*mean* = 7.68; *SE* = 0.23) and the NeuLG (mean = 7.76; SE = 0.23).

### Reproduction of the Eight-Square Sequence on the Outline of the WalCT

Descriptive statistics for this measure follows: *Mean* = 6.32; *SD* = 2.4; *SE* = 0.28; Min = 0–Max = 8.

**FIGURE 4:**
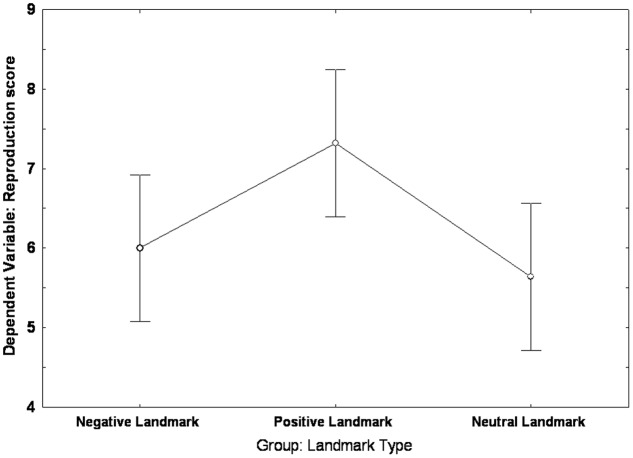
**Landmark group differences in the reproduction of the Walking Corsi Test**. The error bars represent the standard errors of the means (confidence interval = 0.95).

The Univariate ANOVA carried out on the reproduction score revealed an effect of group [*F*(2,72) = 3.6372, *p* = 0.03, partial η^2^ = 0.092]: *Post hoc* analysis (LSD: *p* < 0.05) showed that the PLG (*mean* = 7.32; *SE* = 0.44) scored higher than both the NLG (*mean* = 6; *SE* = 0.44) and the NeuLG (*mean* = 5.64; *SE* = 0.44); no difference was found between the NLG and the NeuLG (See **Figure 4**).

## Discussion

In the present study, the role that emotion plays in shaping our topographical memory was investigated. In general, memory may be enhanced for emotional information (e.g., Cahill and McGaugh, 1998; Hamann et al., 1999; Kensinger and Corkin, 2003). Here, the influence of positive and negative emotional landmarks compared to neutral emotional landmark was investigated in topographical memory as measured using the Walking Corsi Test (WalCT). Two different frames of reference were considered: egocentric coordinates during the learning of an eight-square path and the delayed recall of the same path; and allocentric coordinates during the reproduction of the path on the outline of the WalCT. Indeed, learning and delayed recall of the eight-square path requires the participant to process information about the position of the self relative to the WalCT (egocentric), whereas the reproduction of the learned path on the map requires the participant to process the position of the squares relative to each other in the WalCT (allocentric).

Firstly, results showed that positive and negative emotional landmarks facilitated the learning of the eight-square path as compared to neutral emotional landmarks, confirming, and extending Balaban et al.’s (2017) study, that revealed the key role only of negative emotional landmarks on remembering paths in virtual environments. Contrarily to Balaban et al.’s (2017) study, neutral landmarks were selected with low arousal, whereas both positive and negative emotional landmarks were selected with high arousal. Different studies showed that memory performance is affected by the level of arousal of stimuli, that is high arousal items are better remembered than low arousal items (e.g., Bradley et al., 1992); in addition, arousal was found to enhance memory for high priority information (Mather and Sutherland, 2011). Thus, both positive and negative emotional landmarks with high arousal captured individuals’ attention, producing an improvement of topographical memory, producing a binding of emotional high arousal landmarks with the path. Indeed, when an individual views a highly arousing photo he/she attributes to the photo a distinctiveness that causes the memory enhancement (Ochsner, 2000). At biological level specific stress hormones are released under highly arousing conditions interacting with the amygdala and leading to improvements in memory (McGaugh et al., 2000). In this direction, the amygdala plays a key role in providing attentional advantages to emotional stimuli (Vuilleumier and Driver, 2007), and by consequence emotionally arousing objects attract attention that facilitates binding of their constituent features (Mather, 2007). This explanation was also used by Mather and Nesmith (2008) to account for the enhancement of memory for the location of high arousal pictures. According to these authors, arousal may enhance the binding process of location to arousal stimuli in two ways: increasing the selectivity of attention and increasing the activation level of the features associated with the object (Mather and Nesmith, 2008).

Secondly, no effect of emotional landmarks was found on the delayed recall, whereas positive emotional landmarks enhanced the allocentric topographical memory, facilitating the reproduction of the path on the outline of the WalCT, as compared to both negative and neutral emotional landmarks. In other words, positive emotional landmarks promoted the translation of egocentric information in an allocentric representation more effectively than negative and neutral emotional landmarks, giving rise to a stable survey representation. In this direction, positive high-arousal stimuli (landmarks) are generally recalled more often than negative high arousal stimuli (e.g., Schmidt et al., 2011). Interestingly, positive emotions increase the ability to remember general and heuristic aspects of an experience, enhancing activity within neural regions that support feelings of familiarity (e.g., Levine and Bluck, 2004; Mickley and Kensinger, 2008). According to this interpretation, while building up allocentric representations positive landmarks with high arousal were retrieved from memory more often, enhancing the familiarity of positive landmarks. Iachini et al. (2009) revealed that locations of familiar buildings were mentally represented in terms of allocentric frames of reference, whereas egocentric frames of reference were used when the environment was unfamiliar.

Taken together, these results showed that emotional landmarks (positive or negative) can improve topographical memory, acting as a moderating variable between environment, observer and objects (Caduff and Timpf, 2008). However, a limit of these results has to be found in the experimental set-up used. Indeed, even if WalCT permits a high control on variables and gives general indications about how topographical working memory works (Bianchini et al., 2010), it lacks ecological validity. This means that it should be crucial in the next future to analyze the “real” navigation behavior in ecological environments that include more real emotional landmarks, that are relevant for the individual, such as churches, shops, monuments, cemeteries and so forth. Nevertheless, it is noteworthy to highlight that, learning a path on WalCT is not like learning a path in a real environment it allows to get information about predictive relationship between the WalCT subject’s performance and the subject’s spatial orientation behavior in the real world (e.g., Bianchini et al., 2010; Piccardi et al., 2014).

As a further next step, the issue of the emotional landmarks in navigation should be explored considering not only the general valence of landmarks but also the specific emotions, such as fear, disgust, anger, sadness and so forth for landmarks with negative valence, and relax, love, humor, happiness, and so forth for landmarks with positive valence. In this vein, the arousal issue should also be considered. For example, in light of Gilet and Jallais (2011), critical landmarks could be arranged as follows: on the one hand, positive and negative valence with low arousal (e.g., serenity and sadness); on the other hand, positive and negative valence with high arousal (e.g., happiness and anger). Such conditions would better clarify the unique contribution of valence and arousal to navigation. Finally, the extent to which the issue of navigation and wayfinding can be improved by emotional landmarks should also be investigated in clinical populations with topographical disorientation disorders as well as in patients suffering from mood disorders (i.e., anhedonia in patients suffering from trauma brain injury). In other words, the question is if emotional landmarks can be used as an aid for people that show impairments in navigations.

## Author Contributions

MP planned, arranged the set-up, collected data, and wrote the paper. LP planned and contributed to wrote the paper.

## Conflict of Interest Statement

The authors declare that the research was conducted in the absence of any commercial or financial relationships that could be construed as a potential conflict of interest.
